# Efficacy and Safety of Aidi Injection Combined with Transcatheter Arterial Chemoembolization on Primary Hepatic Carcinoma: A Systematic Review and Meta-Analysis

**DOI:** 10.1155/2018/6376429

**Published:** 2018-07-24

**Authors:** Weihao Chen, Yurong Wang, Qiuer Liang, Yunfei Cai, Xudong Chen, Yun Zhang, Ruixue Chen, Ya Xiao, Liguo Chen

**Affiliations:** Chinese Medicine College, Jinan University, 601 Huangpu West Avenue, Guangzhou 510632, Guangdong, China

## Abstract

**Objectives:**

To evaluate the efficacy and safety of Aidi injection (ADI) combined with transcatheter arterial chemoembolization (TACE) for primary hepatic carcinoma (PHCC).

**Methods:**

We conducted a literature search in EMBASE, PubMed, CENTRAL, MEDLINE, CNKI, Wanfang, and VIP databases from the earliest possible year to April 2018. Randomized controlled trials (RCTs) involving ADI combined with TACE versus TACE alone for patients with PHCC were included. The Cochrane Risk of Bias tool was applied for quality assessment.

**Results:**

22 studies involving 1611 participants were included. The clinical response rate (RR = 1.28, 95% CI: 1.17-1.40;* P* < 0.00001), KPS score (RR = 1.78, 95% CI: 1.59-2.00;* P* < 0.00001), survival rate (RR = 1.27, 95% CI: 1.16-1.39;* P* < 0.00001), immune function (MD = 1.24, 95% CI: 0.98-1.51;* P* < 0.00001), and adverse effects (RR = 0.62, 95% CI: 0.57-0.68;* P* < 0.00001) of ADI plus TACE showed significant difference when compared with TACE alone.

**Conclusions:**

ADI combined with TACE in the treatment of PHCC improved the clinical response rate and safety compared to TACE alone. However, due to poor methodological quality of many of the included RCTs, more rigorously designed and large-scale RCTs are warranted to examine this beneficial effect in the future.

## 1. Introduction

Primary hepatic carcinoma (PHCC) is the 5th most common malignant tumor of digestive system in the world, which accounts for 90% of its pathological type. Moreover, PHCC is the 3rd contributor to the cause of cancer-related death [1, 2]. More than 500,000 people in the world suffered from PHCC every year, and 55% of them are in China [3, 4]. At present, the methods in the treatment of PHCC were surgery, hepatic artery ligation, liver transplantation, transcatheter arterial chemoembolization (TACE), radiofrequency ablation, cryotherapy, laser, and biological therapy. For now, surgery or liver transplantation is an effective treatment for early PHCC, but due to the rapid progression and concealment of PHCC, symptoms are not obvious or there were no symptoms in early stage. 70%-80% patients with PHCC have been diagnosed as advance or distant metastasis when they visited the doctor [5]. In addition, many patients of PHCC had severe complication of cirrhosis which results in an inability to undergo the surgical treatment and poor prognosis.

TACE is a minimally invasive interventional radiology which is an important method in the treatment which inhibits tumor growth and promotes tumor cell necrosis and apoptosis in PHCC via applying antitumor drug to block the blood supply, resulting in tumor ischemia and hypoxia. Currently, TACE has been widely applied to patients with PHCC who were not suitable for surgery in advance stage [6] or used as an alternative to early resection of PHCC and in patients with recurrence after tumor resection. Therefore, TACE has become the first choice for the treatment of PHCC in recent years. However, in the meantime, TACE has a lot of disadvantages of chemotherapy-induced adverse reactions, such as gastrointestinal reaction, blood toxicity, bone marrow suppression, hepatotoxicity, and nephrotoxicity [7]. The adverse effects may further affect the recovery and prognosis of patient with PHCC.

ADI was refined from four Traditional Chinese Medicines (TCM) by modern scientific methods which consist of* Ginseng* (Rensheng),* Spanish fly* (Banmou),* Astragalus* (Huangqi), and* Acanthopanax senticosus* (Ciwujia). In China, ADI combined with TACE has been widely applied in the clinical treatment of PHCC. Many RCTs have demonstrated that ADI can effectively improve immunity, reduce adverse effects of TACE, and reduce the recurrence and metastasis of patients with PHCC [8]. However, the treatment of ADI combined with TACE for the PHCC still lacks systematic evaluation criteria. Therefore, this meta-analysis is aimed to investigate whether ADI combined with TACE can improve clinical response rate, KPS scores, and survival rate, enhance function of immune, and reduce adverse effects in the treatment of patients with PHCC compared to TACE alone.

## 2. Methods

### 2.1. Type of Studies

Our study included all of the RCTs reporting ADI combined with TACE in the treatment of PHCC.

### 2.2. Type of Participants

Only patients with a diagnosis of PHCC based on “Standard for diagnosis and treatment of common malignant tumors in China” (2011 edition) were included. There were no limitations on age and gender.

### 2.3. Type of Interventions

Patients in the experiment group were given ADI plus TACE. And patients in the control group were given TACE only. The experiment group or control group which included other interventions were excluded. There were no limitations on dosage and treatment cycle of ADI and TACE.

### 2.4. Type of Outcome Measures

The primary outcomes were clinical response rate and Karnofsky Performance Status (KPS). The secondary outcomes were survival rate (6 months survival rate, 12 months survival rate, and 24 months survival rate), immune function (CD^3+^, CD^4+^, CD^8+^, and CD^4+^/CD^8+^), and adverse effects (WBC reduction, gastrointestinal reaction, bone marrow suppression, fever, liver function, and Child-Pugh). Clinical response rate is the combined rate of CR (complete response) and PR (partial response) as defined by the WHO and determined by imaging. We calculate the number of patients with improved performance status (more than 10 KPS points increase) after treatment.

### 2.5. Literature Search

Our meta-analysis was conducted according to the Preferred Reporting Items of Systematic reviews and Meta-Analyses (PRISMA) guidelines. We indecently conducted a comprehensive literature search in EMBASE, PubMed, CENTRAL, MEDLINE, CNKI, Wanfang, and VIP databases from the earliest possible year to April 2018 and no language restrictions. We used various combinations of Mesh headings and keywords to form the following search terms: (((primary liver cancer) OR (primary cancer of liver) OR (primary hepatic neoplasm) OR (primary hepatocellular cancer) OR (primary hepatocellular carcinoma)) and ((Aidi injection) OR (Aidi) OR (Aidi Zhusheye)) and ((transcatheter arterial chemoembolization) OR (TACE))). In addition, we also manually searched the references cited for relevant studies.

### 2.6. Data Extraction

Two reviewers (Weihao Chen and Yurong Wang) independently assessed the included studies by examined titles and abstracts and excluded the studies which did not meet the inclusion criteria. To avoided subjectivity, the authors' name and institution were blinded to reviewer. We resolved all the disagreement by discussed with the third reviewer (Liguo Chen). The following information from each included study was extracted: first author, publication year, sample size, patients' age, cancer stage, intervention detail, treatment course, KPS scores, adverse effects, and outcome measures.

### 2.7. Study Quality Evaluation

Two reviewers (Qiuer Liang and Xudong Chen) evaluated the risk of bias of included studies according to the Cochrane Risk of Bias tool [9] which is based on six aspects: (1) selection bias (random sequence generation and allocation concealment); (2) performance bias (binding of participants and personnel); (3) detection bias (blinding of outcome assessment); (4) attrition bias (incomplete outcome date); (5) reporting bias (selective reporting); and (6) other bias (other potential bias). We resolved all the disagreements by discussing with third author (Liguo Chen) to reach consensus.

### 2.8. Data Analysis

We used the Review Manager (RevMan) Program (Version 5.3.5 Copenhagen: The Nordic Cochrane Centre, The Cochrane Collaboration, 2014) and Stata/SE version 14.0 software (Stata Corporation, College Station, Tex) to pool and analyze data. We calculated the mean differences (MD) and relative risk (RR) with 95% confidence intervals (CI) to compare continuous and dichotomous variables, respectively. The heterogeneity of included studies was calculated by Cochran's Q-statistic and* I*^*2*^-statistic [10, 11]. If significant heterogeneity was present (*I*^*2*^ ≥ 50% and* P* < 0.05), the random-effects model was used to synthesize the data. Otherwise, the fixed-effects model was applied. We utilized the funnel plots and Egger's test to evaluate the publication bias if more than 10 studies were included [12].

## 3. Results

### 3.1. Study Selection

**Figure 1:**
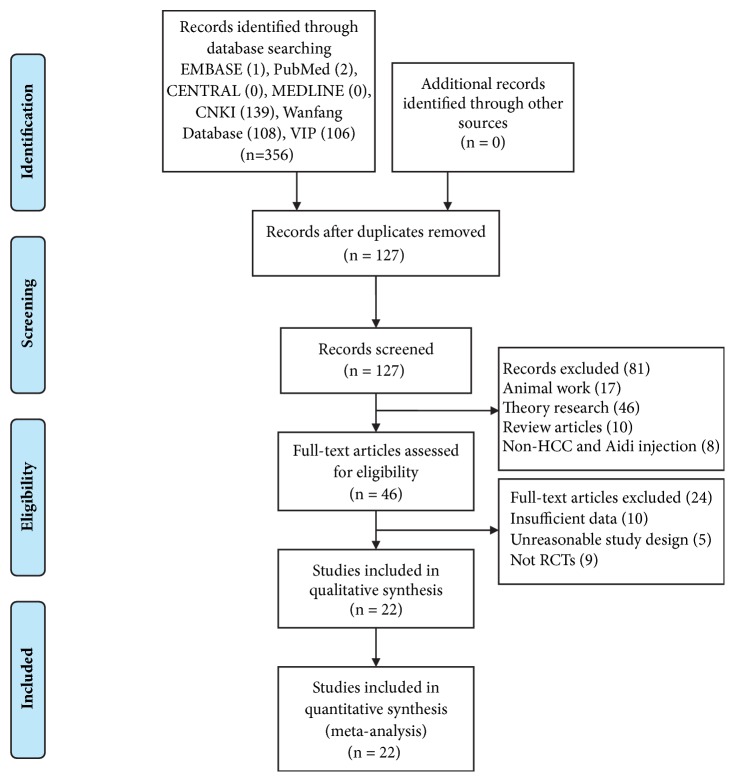
Flow diagram of study selection.

Our literature search yielded 356 studies in EMBASE, PubMed, CENTRAL, MEDLINE, CNKI, Wanfang, and VIP databases and manual search. Screened on the basis of title and abstract, a total of 127 articles were retrieved after removing duplicates. Then we excluded 81 articles which did not meet our inclusion criteria, including 17 animal articles, 46 theory research articles, 8 non-HCC and ADI articles, and 10 review articles. 46 articles were assessed with full-text. After that, we excluded 24 articles because of the following reasons: 10 insufficient data articles, 5 unreasonable study design articles, and 9 non-RCTs articles. Finally, a total of 22 studies [13–34] remained and were included in our meta-analysis (Figure 1).

### 3.2. Study Characteristics

The total number of participants in this meta-analysis was 1611 (818 in the experiment group and 793 in the control group), with an age range from 28 to 75. All the studies originated from China and published in Chinese and involved two-arm design: experiment group versus control group. Experiment group was given ADI plus TACE treatment and control group was given TACE treatment only. All the studies reported clinical response rate and KPS, 7 studies reported survival rate [18, 23, 25, 28, 29, 31, 33], 4 studies described immune function [18, 20, 22, 31], and 14 articles discussed adverse effects [13–18, 21–23, 25, 26, 28, 29, 33].  Table 1 presents the basic information and detailed characteristics of the 22 included studies.

### 3.3. Primary Outcomes

#### 3.3.1. Clinical Response Rate

**Figure 2:**
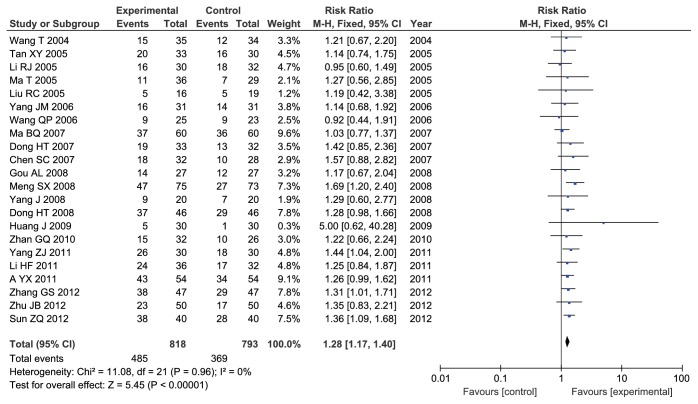
Forest plot of improved clinical response rate.

All studies including 1611 participants reported clinical response rate. No heterogeneity between-study was observed (Chi^2^ = 11.08,* I*^*2*^ = 0%,* P* = 0.96). The fixed-effects model was applied to analysis. The results showed that ADI combined with TACE significantly improved the clinical response rate of patients with PHCC when compared with TACE alone (RR = 1.28, 95% CI: 1.17-1.40;* P* < 0.00001) (Figure 2).

#### 3.3.2. KPS Score Evaluation

**Figure 3:**
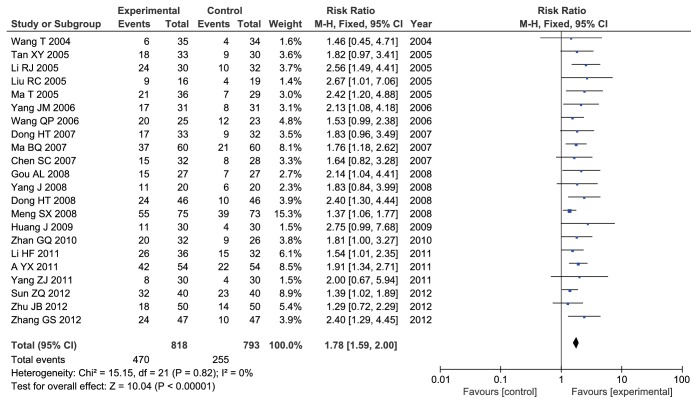
Forest plot of KPS score.

All studies including 1611 participants assessed KPS scores. There was no heterogeneity between-study (Chi^2^ = 15.15,* I*^*2*^ = 0%,* P* = 0.82). Therefore, we used fixed-effects models to calculate combined RR and 95% CI. The results showed that there was a statistically significant difference between experiment and control group, and ADI combined with TACE significantly increase KPS scores to improve patients' quality life with PHCC when compared with TACE alone (RR = 1.78, 95% CI: 1.59-2.00;* P* < 0.00001) (Figure 3).

### 3.4. Secondary Outcomes

#### 3.4.1. Survival Rate

**Figure 4:**
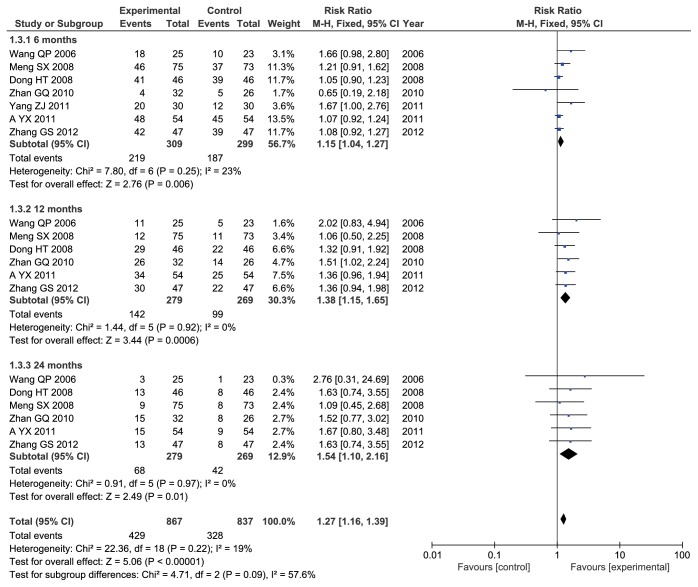
Forest plot of survival rate.

Seven studies [18, 23, 25, 28, 29, 31, 33] involved 608 participants and reported 6-month survival rate. No heterogeneity between-study was observed (Chi^2^ = 7.8,* I*^*2*^ = 23%,* P* = 0.25). The fixed-effects model was applied to analysis. The results showed that ADI combined with TACE significantly improved the 6-month survival rate of patients with PHCC when compared with TACE alone (RR = 1.15, 95% CI: 1.04-1.27;* P* = 0.006) (Figure 4).

Six studies [18, 23, 25, 28, 29, 33] contain 548 patients and described 12-month survival rate. There was no heterogeneity between-study (Chi^2^ = 1.44,* I*^*2*^ = 0%,* P* = 0.92). We used fixed-effects models to synthesized RR and 95% CI. The results indicated that there was a statistically significant difference between two groups and ADI combined with TACE significantly improved the 12-month survival rate of patients with PHCC when compared with TACE alone (RR = 1.38, 95% CI: 1.15-1.65;* P* = 0.0006) (Figure 4).

Six studies [18, 23, 25, 28, 29, 33] including 548 patients reported 24-month survival rate. No heterogeneity between-study was observed (Chi^2^ = 0.91,* I*^*2*^ = 0%,* P* = 0.97). The fixed-effects model was applied to analysis. The results indicated that there was a statistically significant difference between two groups and ADI combined with TACE significantly improved the 24-month survival rate of patients with PHCC when compared with TACE alone (RR = 1.54, 95% CI: 1.10-2.16;* P* = 0.01) (Figure 4).

A total of 7 [18, 23, 25, 28, 29, 31, 33] studies with 1704 cases reported survival rate. No heterogeneity between-study was observed (Chi^2^ = 22.36,* I*^*2*^ = 19%,* P* = 0.22). The fixed-effects model was applied to synthesized data. The results indicated that there was a statistically significant difference between two groups and ADI combined with TACE significantly improved the survival rate of patients with PHCC when compared with TACE alone (RR = 1.27, 95% CI: 1.16-1.39;* P* < 0.00001) (Figure 4).

#### 3.4.2. Immune Function

**Figure 5:**
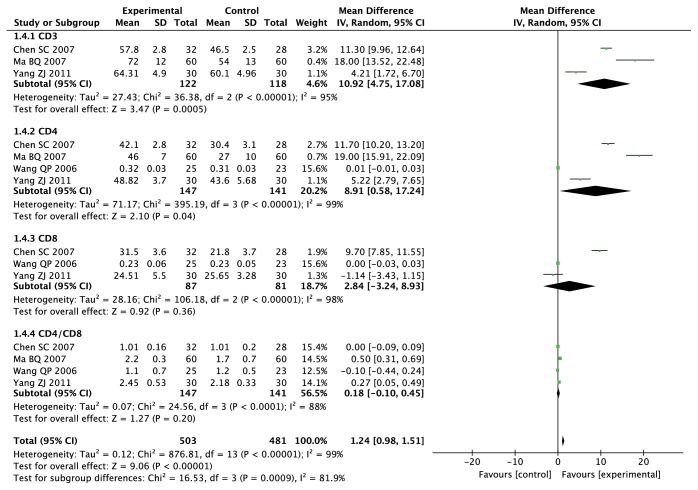
Forest plot of immune function.

Four studies [18, 20, 22, 31] including 288 cases reported the expression level of CD^3+^ which is a biomarker of immune function. The result of heterogeneity test showed that there was significant evidence of heterogeneity between-study (Chi^2^ = 36.38,* I*^*2*^ = 95%,* P* < 0.00001). So we used random-effects models to synthesized MD (Mean Difference) and 95% CI. The results indicated that there was a statistically significant difference between two groups and ADI combined with TACE group significantly increase CD^3+^ expression when compared with TACE alone (MD = 10.92, 95% CI: 4.75-17.08;* P* = 0.0005) (Figure 5).

The expression levels of CD^4+^ were reported in 4 studies [18, 20, 22, 31] which involved 288 cases. The result of heterogeneity test showed that there was significant evidence of heterogeneity between-study (Chi^2^ = 395.19,* I*^*2*^ = 99%,* P* < 0.00001). Therefore, we used random-effects models to calculate MD and 95% CI. The results indicated that there was a statistically significant difference between two groups and ADI combined with TACE significantly increase CD^4+^ expression when compared with TACE alone (MD = 8.91, 95% CI: 0.58-17.24;* P* = 0.04 (Figure 5).

Three studies [18, 20, 31] including 168 patients reported the expression level of CD^8+^. The result of heterogeneity test showed that there was significant evidence of heterogeneity between-study (Chi^2^ = 106.18,* I*^*2*^ = 98%,* P* < 0.00001). Random-effects model was applied to analysis. The results showed that there was no statistical difference between two groups and ADI combined with TACE group did not affect CD^8+^ expression (MD = 2.84, 95% CI: -3.24-8.93;* P* = 0.36) (Figure 5).

Four studies [18, 20, 22, 31] including 288 patients reported the expression level of CD^4+^/CD^8+^. The result of heterogeneity test showed that there was significant evidence of heterogeneity between-study (Chi^2^ = 24.56,* I*^*2*^ = 88%,* P* < 0.0001). Random-effects model was applied to analysis. The results showed that there was no statistical difference between two groups and ADI combined with TACE group did not affect the expression level of CD^4+^/CD^8+^ (MD = 0.18, 95% CI: -0.10-0.45;* P* = 0.2) (Figure 5).

A total of 4 studies [18, 20, 22, 31] with 984 cases reported immune function. There was significant evidence of heterogeneity between-study (Chi^2^ = 876.81,* I*^*2*^ = 99%,* P* < 0.00001). Random-effects model was applied to analysis. The results showed that there was a statistically significant difference between two groups and ADI combined with TACE significantly improve immune function and reduce blood toxicity of patients with PHCC when compared with TACE alone (MD = 1.24, 95% CI: 0.98-1.51;* P* < 0.00001) (Figure 5).

#### 3.4.3. Adverse Effects

**Figure 6:**
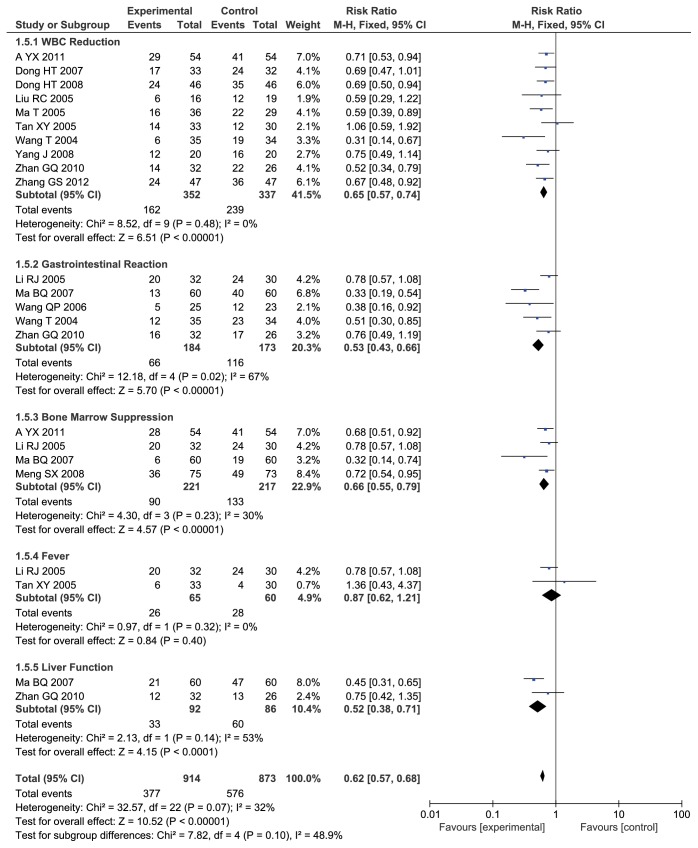
Forest plot of adverse effects.

Ten studies with 689 cases reported white blood cell (WBC) reduction after treatment. No heterogeneity between-study was observed (Chi^2^ = 8.52,* I*^*2*^ = 0%,* P* = 0.48). The results showed that ADI combined with TACE significantly improved the WBC expression of patients with PHCC when compared with TACE alone (RR = 0.65, 95% CI: 0.57-0.74;* P* < 0.00001) (Figure 6).

Five studies including 357 cases reported gastrointestinal reaction. The result of heterogeneity test showed that there was some evidence of heterogeneity between-study (Chi^2^ = 12.18,* I*^*2*^ = 67%,* P* = 0.02). And the results showed that there was a statistically significant difference between two groups and ADI combined with TACE significantly improved the gastrointestinal reaction of patients with PHCC when compared with TACE alone (RR = 0.53, 95% CI: 0.43-0.66;* P* < 0.00001) (Figure 6).

Four studies with 438 cases reported bone marrow suppression. No heterogeneity between-study was observed (Chi^2^ = 4.30,* I*^*2*^ = 30%,* P* = 0.23). The results showed that there was a statistically significant difference between two groups and ADI combined with TACE significantly improved the bone marrow suppression of patients with PHCC when compared with TACE alone (RR = 0.66, 95% CI: 0.55-0.79;* P* < 0.00001) (Figure 6).

Two studies with 125 cases reported adverse effects about fever. No heterogeneity between-study was observed (Chi^2^ = 0.97,* I*^*2*^ = 0%,* P* = 0.32). The results showed that there was no statistically difference between two groups and ADI combined with TACE did not improve the fever of patients with PHCC (RR = 0.87, 95% CI: 0.62-1.21;* P* = 0.4) (Figure 6).

Two studies with 178 cases reported liver function. There was a little of heterogeneity between-study (Chi^2^ = 2.13,* I*^*2*^ = 53%,* P* = 0.14). And the results indicated that there was a statistically significant difference between two groups and ADI combined with TACE significantly improved the liver function of patients with PHCC when compared with TACE alone (RR = 0.52, 95% CI: 0.38-0.71;* P* < 0.0001) (Figure 6).

A total of 14 [13–18, 21–23, 25, 26, 28, 29, 33] studies with 1787 cases reported adverse effects. No heterogeneity between-study was observed (Chi^2^ = 32.57,* I*^*2*^ = 32%,* P* = 0.07). The results indicated that there was a statistically significant difference between two groups and ADI combined with TACE significantly improved the adverse effects of patients with PHCC when compared with TACE alone (RR = 0.62, 95% CI: 0.57-0.68;* P* < 0.00001) (Figure 6).

### 3.5. Risk of Bias

**Figure 7:**
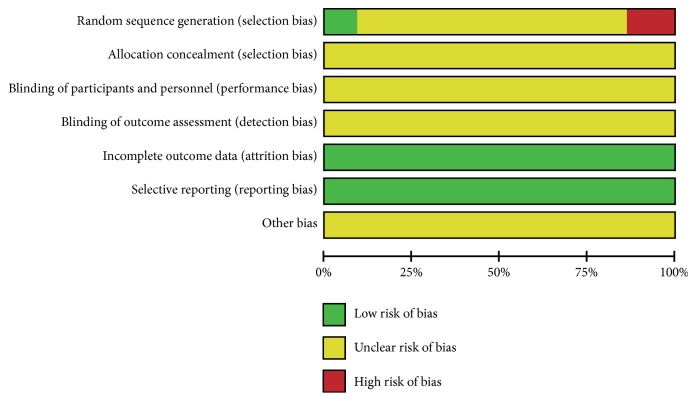
Risk of bias graph.

**Figure 8:**
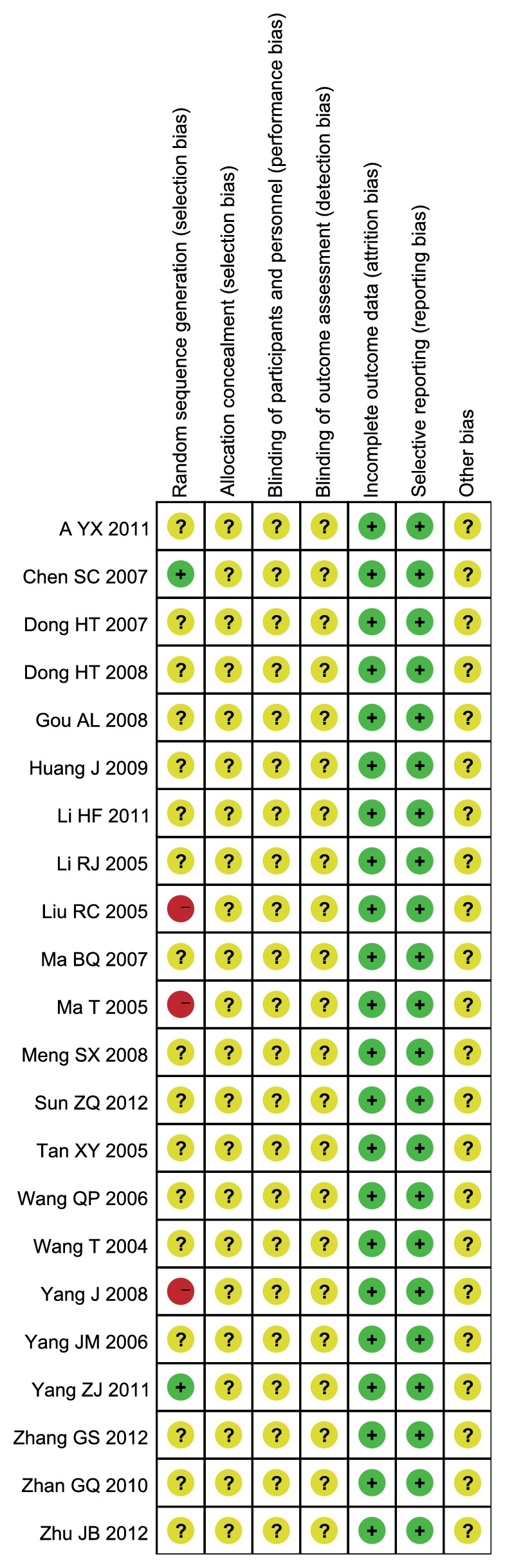
Risk of bias summary of included studies.

Although 17 of 22 included studies [13, 14, 17, 19–25, 27–33] described the randomization, no methods of randomization were mentioned. Only 2 articles [18, 26, 34] described the method of randomization and 3 studies [15, 16, 26] reported false method of randomization. None of studies reported allocation concealment, blinding of participants, and personnel and outcome assessment. All studies provide complete outcome data and no selective reporting. It is hard to judge whether there are other sources of bias, so we marked the other bias as unclear risk (Figures 7 and 8).

### 3.6. Publication Bias

**Figure 9:**
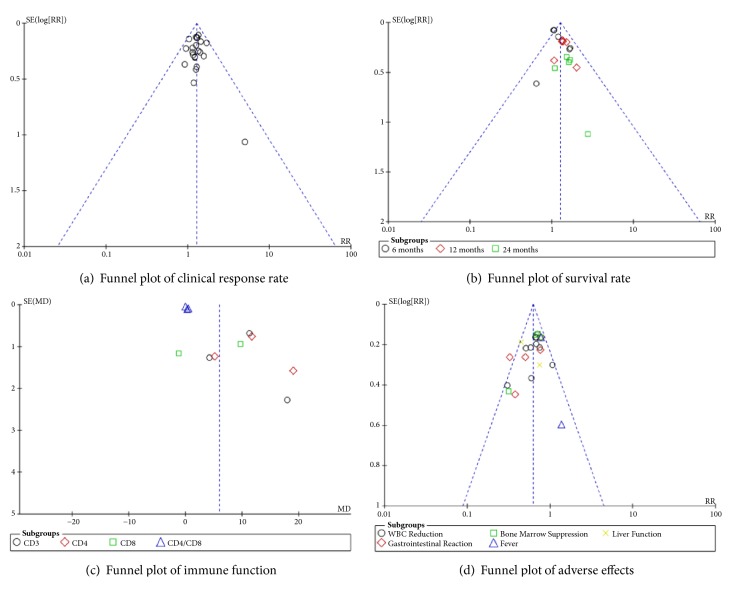
Funnel plot.

We included sufficient studies in this systematic review so we can make a funnel plot and Egger's test for publication bias of clinical response rate, survival rate, immune function, and adverse effects. Based on the Egger's testing results, we found that there was potential publication bias in “survival rate” (*P* = 0.001 < 0.05) and “immune function” (*P* = 0.002 < 0.05), while potential publication bias in “clinical response rate” (*P* = 0.962 > 0.05) and “adverse effects” (*P* = 0.093 > 0.05) was not (Figure 9).

## 4. Discussion

### 4.1. Summary of Main Results

TACE is effective in the treatment of unresectable primary hepatocellular carcinoma, but traumatic treatment and adverse effects of anticancer drugs also affect the patient's survival and quality of life. As a common TCM reagent with operation and radiochemotherapy, ADI has obvious advantages in enhancing efficacy, reducing toxicity, improving quality of life, and prolonging survival period of PHCC patients. In this meta-analysis, based on 22 included studies, our finding indicated that ADI combined with TACE significant improved clinical response rate and increased KPS scores, expression level of CD^3+^ and CD^4+^, improved survival rate of 6 moths, 12 months, 24 months, and adverse effects of WBC reduction, gastrointestinal reaction, bone marrow suppression, and liver function of patients with PHCC when compared with TACE alone. However, the results of the expression level of CD^8+^, CD^4+^/CD^8+^, and adverse effect of fever showed that there is no statistically difference between experiment group and control group.

### 4.2. Analysis of Aidi Injection

ADI as adjuvant TACE has been widely used in the treatment of primary hepatocellular carcinoma. It was approved by the Ministry of Health's drug standard of Chinese Materia Medica preparation (20th volume) and National Drug Standard (revised) in 2002 (Standard Number: WS3-B-3809-99-2002). In modern medical research, there are several biologic mechanisms to explain the protective effects of ADI on the patients with PHCC. For example, pharmacology study has shown that ADI contained a variety of polysaccharides including* astragalus *and* acanthopanax senticosus* which is refined and extracted by modern scientific methods. This can improve the phagocytosis of reticuloendothelial system, stimulate the production of TNF-*α* (tumor necrosis factor), and enhance the activity of T cells, NK cells, and Lak (lymphoid activated killer cells) of PHCC patients [35–37]. Furthermore, some researchers found that* Ginseng (Rensheng) *contains Rg3 and RH2 in Ginsenoside which can also enhance and improve the function of T cell and B cell, increase a number of interferon and interleukin, and enhance the killing ability of NK (natural killer) cells and lymphatic factor [38]. An additional underlying mechanism is that cantharidin may inhibit the synthesis of protein, downregulate the activation level of oncogene, and affect the nucleic acid metabolism in cancer cells via the interference of cell proliferation and induction of apoptosis [39, 40] and norcantharidin promote the apoptosis of tumor cells and inhibit the angiogenesis of tumor [41, 42] which both are components of* Spanish fly (Banmou). *In addition,* Ginseng *(Rensheng) and* Astragalus *(Huangqi) have the effect of tonifying QI.* Spanish fly *(Banmou) and* Acanthopanax senticosus *(Ciwujia) have the effect of clearing away heat and toxic materials and dissipating mass in the theory of traditional Chinese medicine [8]. Therefore, combining the above four kinds of herbs, ADI can greatly enhance the ability of Fuzheng Guben. In other words, it can enhance the function of immunity against PHCC.

### 4.3. Limitations

The meta-analysis is the first system review about ADI combined with TACE in the treatment of patients with PHCC. The advantages of our meta-analysis included many specific outcomes of clinical response rate, KPS scores, survival rate (6, 12, and 24 months), immune function (CD^3+^, CD^4+^, CD^8+^, and CD^4+^/CD^8+^), and adverse effects (WBC reduction, gastrointestinal reaction, bone marrow suppression, fever, and liver function) for compared ADI plus TACE with TACE alone. However, there were several limitations in our meta-analysis. First, the methodological quality of the included studies was generally poor. Although most of included studies described are randomized, there were three studies [15, 16, 26] which described the false method of random sequence generation. Only two trials [20, 31] reported the right method of random sequence generation. None of included studies described allocation concealment and blinding of participants, and personnel and outcome assessment. Second, although we found that ADI plus TACE has a better protective effect on PHCC patients when compared with TACE alone, we should be interpreted with caution because there was the existence of heterogeneity (immune function) and potential publication bias by visual asymmetry from funnel plot and Egger's test. To explore the heterogeneous sources of immune function, we conducted subgroup analysis based on CD^3+^, CD^4+^, CD^8+^, and CD^4+^/CD^8+^. The results showed that each subgroup had distinct heterogeneity (CD^3+^:* I*^*2*^ = 95%, CD^4+^:* I*^*2*^ = 96%, CD^8+^:* I*^*2*^ = 98%, CD^4+^/CD^8+^:* I*^*2*^ = 91%). And methodological heterogeneity may be one of the heterogeneity sources. Third, all the included studies were published in Chinese which might lead to ethnic bias. Moreover, after a comprehensive search of databases, the information of ADI with other drugs interaction is not available. In the future, it is necessary for us to conduct research in this area.

## 5. Conclusion

This meta-analysis indicated that Aidi injection combined with TACE in the treatment of primary hepatocellular carcinoma improved the clinical response rate and safety compared to TACE alone. However, due to poor methodological quality of many of the included RCTs, more rigorously designed, multicenter, large sample, RCTs are warranted to examine this beneficial effect before drawing definitive conclusions. In the meantime, it is reasonable for patients to consider Aidi injections alongside TACE in the interim, but high quality studies should be conducted to confirm benefit.

## Figures and Tables

**Table 1:** Characteristics of the 22 included studies.

First author, year	Sample size (E/C)	Age(E/C)	Stage	Intervention^†^ (E/C)	Treatment course (C/W/D)	KPS	Outcome
Wang T, 2004	35/34	65.8/66.7	II-III	ADI+TACE(MEF)	1C, 30D/C	>60	①②⑤
Tan XY, 2005	33/30	38	-	ADI+TACE(DM“E-A/L”F)	2C, 30-40D/C	≥60	①②⑤
Li RJ, 2005	32/30	53	-	ADI+TACE(DELF)	2C, 3-4W/C	≥60	①②⑤
Ma T, 2005	36/29	50/52	I-III	ADI+TACE(DHLF)	2C, 3-4W/C	>60	①②⑤
Liu RC, 2005	16/19	52/49	I-III	ADI+TACE(DALF)	2C, 4-5W/C	>60	①②⑤
Yang JM, 2006	31/31	49	-	ADI+TACE(DELF)	2C, 4-12W/C	-	①②
Wang QP, 2006	25/23	50.1	-	ADI+TACE(M“A/E”LF)	3C, 4-5W/C	-	①②③④⑤
Ma BQ, 2007	60/60	44	-	ADI+TACE(DCMLF)	3C, 35D/C	≥60	①②④⑤
Dong HT, 2007	33/32	56.3/56.7	II-IV	ADI+TACE(TLF)	2C, 28D/C	≥60	①②⑤
Chen SC, 2007	32/28	36-70	-	ADI+TACE(MOLF)	2-6C, 4-6W/C	>60	①②④
Gou AL, 2008	27/27	49	-	ADI+TACE(DELF)	2C, 4-12W/C	-	①②
Meng SX, 2008	75/73	55.2/56.8	II-IV	ADI+TACE(TLF)	2C, 2W/C	>60	①②③⑤
Yang J, 2008	20/20	28-74	-	ADI+TACE(D“A/M”LF)	2-3C, 28D/C	>40	①②⑤
Dong HT, 2008	46/46	56±3/55.7±3	II-IV	ADI+TACE(HLF)	2C, 28D/C	>60	①②③⑤
Huang J, 2009	30/30	45.22/44.98	-	ADI+TACE(DELF)	2C, 3D/C	30-60	①②
Zhan GQ, 2010	32/26	43.6	II-III	ADI+TACE(MHELF)	2C, 3-4W/C	>50	①②③⑤
Yang ZJ, 2011	30/30	49.8/49	-	ADI+TACE(M“H/E”LF)	3C, 4-6W/C	≥70	①②③④
Li HF, 2011	36/32	52±4	-	ADI+TACE(MTLF)	1C, 12W/C	≥70	①②
A YX, 2011	54/54	56.3/55.7	II-IV	ADI+TACE(HLF)	2C, 28D/C	≥60	①②③⑤
Zhang GS, 2012	47/47	57.1±3/56.7±3	II-IV	ADI+TACE(HLF)	2C, 28D/C	≥60	①②③⑤
Zhu JB, 2012	50/50	35-75	-	ADI+TACE(OLT)	2-5C, 4-6W/C	>60	①②
Sun ZQ, 2012	40/40	56.2	-	ADI+TACE(OHEL)	2-4C, 4-6W/C	-	①②

Note: E/C: experimental group/control group; C: cycle; W: week; D: day; KPS: Karnofsky; intervention: TACE (transcatheter arterial chemoembolization); ADI: Aidi injection; M: MMC (mitomycin); E: EPI (epirubicin); F: 5-FU (5-fluorouracil); A: ADM (adriamycin); L: LUF (lipiodol ultra fluid); D: DDP (cisplatin); H: HCPT (hydroxycamptothecin); T: THP (tetrahydropyranyl); C: CF (calcium folinate); O: OXA (oxaliplatin); outcome: ①clinical response rate; ②KPS; ③survival rate; ④immune function; and ⑤adverse effects. ^†^The experiment group was treated with ADI and TACE, and control group was treated with TACE alone.

## Data Availability

The data used to support the findings of this study are included within the article.
